# A Longitudinal Study of Household Water, Sanitation, and Hygiene Characteristics and Environmental Enteropathy Markers in Children Less than 24 Months in Iquitos, Peru

**DOI:** 10.4269/ajtmh.17-0464

**Published:** 2018-02-12

**Authors:** Natalie G. Exum, Gwenyth O. Lee, Maribel Paredes Olórtegui, Pablo Peñataro Yori, Mery Siguas Salas, Dixner Rengifo Trigoso, Josh M. Colston, Kellogg J. Schwab, Benjamin J. J. McCormick, Margaret N. Kosek

**Affiliations:** 1 Department of Environmental Health and Engineering, Bloomberg School of Public Health, Johns Hopkins University, Baltimore, Maryland;; 2 Department of Epidemiology, University of Michigan, Ann Arbor, Michigan;; 3 Department of International Health, Johns Hopkins Bloomberg School of Public Health, Baltimore, Maryland;; 4 Asociación Benéfica Proyectos de Informática, Salud, Medicina, y Agricultura (A.B. PRISMA), Iquitos, Peru;; 5 Fogarty International Center/National Institutes of Health, Bethesda, Maryland

## Abstract

Poor child gut health, resulting from a lack of access to an improved toilet or clean water, has been proposed as a biological mechanism underlying child stunting and oral vaccine failure. Characteristics related to household sanitation, water use, and hygiene were measured among a birth cohort of 270 children from peri-urban Iquitos Peru. These children had monthly stool samples and urine samples at four time points and serum samples at (2–4) time points analyzed for biomarkers related to intestinal inflammation and permeability. We found that less storage of fecal matter near the household along with a reliable water connection were associated with reduced inflammation, most prominently the fecal biomarker myeloperoxidase (MPO) (no sanitation facility compared with those with an onsite toilet had −0.43 log MPO, 95% confidence interval [CI]: −0.74, −0.13; and households with an intermittent connection versus those with a continuous supply had +0.36 log MPO, 95% CI: 0.08, 0.63). These results provide preliminary evidence for the hypothesis that children less than 24 months of age living in unsanitary conditions will have elevated gut inflammation.

## INTRODUCTION

Environments with high fecal contamination put children at risk for chronic exposure to enteric pathogens, especially in the developing world where an estimated 4.5 billion people lack access to safely managed sanitation.^1^ Frequent episodes of acute gastroenteritis and subclinical enteropathogen infections may lead to persistent inflammation and structural changes in the small bowel, a condition known as environmental enteropathy (EE).^2^ The development of EE during the critical growth period of < 2 years of age may lead to irreversible linear growth deficits, oral vaccine failure, and a loss of human capital from reduced cognitive achievement.^3–5^

Noninvasive fecal biomarkers analyzed in the Etiology, Risk Factors, and Interactions of Enteric Infections and Malnutrition and the Consequences for Child Health and Development Project (MAL-ED) study are shown to be inversely associated with growth.^6,7^ These biomarkers in stool included myeloperoxidase (MPO) and neopterin (NEO), both chosen as markers of intestinal inflammation to represent immune activation, and alpha-1-antitrypsin (AAT), a marker of intestinal permeability and mucosal protein wasting secondary to EE.^8,9^ And in urine, a dual sugar test for intestinal permeability was used to measure the ratio of lactulose to mannitol, whereas in plasma, the biomarkers measured included alpha-1-acid glycoprotein (AGP) as a marker for systemic inflammation, citrulline for overall mucosal function,^10^ and kynurenine, tryptophan, and the kynurenine to tryptophan ratio as markers for immunomodulation occurring from intestinal injury and inflammation.^11^

It is now recognized that asymptomatic enteric infections are frequent and have impacts on the health and development of children.^12^ Therefore, measuring the impact of water, sanitation, and hygiene (WASH) interventions using acute gastroenteritis may be unsatisfactory. Biomarkers for EE may be useful as an alternative means of measuring the long-term developmental impact of WASH interventions as they can be measured noninvasively and cross-sectionally. Recent evidence from the MAL-ED study found that a lower socioeconomic index score (inclusive of water and sanitation, assets, maternal education, and income [WAMI]) was associated with increased EE fecal biomarkers (MPO and AAT), although none of the individual components of the score were consistently associated across sites.^13^ Given that the etiologies and risk factors for EE may differ in different populations, as well as interact with each other in a contextually specific manner, it has been suggested that the fecal biomarkers may be useful in characterizing these risk factors in a site or population-specific manner.^6^

This study investigates the associations between household WASH characteristics and biomarkers for EE in a longitudinal cohort of children less than 24 months of age at the Iquitos, Peru site, of the MAL-ED study. We hypothesized that reasonable improvements in water and sanitary infrastructure and hygienic practices, as they related to fecal–oral pathogen transmission, could improve the small intestine structure and function. A comprehensive set of WASH variables, along with an in-depth characterization of water storage practices, is examined in relation to fecal biomarkers for EE (along with urine and plasma markers).

## MATERIALS AND METHODS

### Study site and population.

The study site is located along the Nanay River, a tributary of the Amazon, in three peri-urban communities—Santa Clara de Nanay, Santo Tomas, and La Union (3°47′S, 73°20′W). These communities are located about 15 km outside of the city center of Iquitos and have a combined population of approximately 5,000 people and a population density of 4.6 people per square meter.^14^ Despite Peru’s success in meeting its Millennium Development Goals for both access to improved water and sanitation,^15^ these peri-urban communities still lag behind the country averages with only 50% of the population using an improved water source and 20% with access to an improved toilet facility.^14^ There is no centralized sewerage in the community, and therefore, even those that have an improved pit latrine lack services to hygienically empty, transport, and treat fecal matter. The community is vulnerable to frequent floods that inundate latrines causing overflow and putting those with onsite sanitation at greatest risk for contamination. Water storage risks recontamination of improved drinking supplies^16^ and is used widely throughout the community because of the intermittent supply for those connected to the piped system and frequent breakdowns of hand pumps.

Childhood stunting is remarkably high in this study community when compared with the rest of Peru and beyond. For children less than 5 years old in peri-urban Iquitos, Peru, 46.3% are stunted (HAZ < −2)^14^ compared with Africa and Asia where 35.6% and 26.8% of children less than 5 years old are stunted, respectively.^17^ A cohort study in Santa Clara found the incidence of diarrheal illness in children 12–23 months of age is 4.38 episodes per child-year,^18^ which is relatively high compared with rates reported in the literature for the last decade.^19^ The number of pathogens detected in the stool of children less than two years old at the Peru site was low for children at 3 months old relative to other MAL-ED sites (about 0.5 pathogens detected per stool). The Peruvian children then acquired more pathogens per stool and by 24 months old the Peru site was in the high range with about 2.0 pathogens detected per stool.^12^

### Selection criteria.

The MAL-ED birth cohort used a prospective longitudinal design among eight sites with historically high incidence of diarrheal disease and undernutrition.^20^ Approximately 200 healthy infants born to mothers greater than 16 years old were enrolled within 17 days of birth. Enrollment was limited to one child per household and children were excluded from the cohort if they were enrolled for less than 6 months, had a caregiver with plans to move out of the catchment area during the first 6 months of follow-up, exhibited serious indications of disease, or were of low birth weight (< 1,500 g). Enrollment occurred over a two year period from January 2010 to February 2012 and children were followed through 24 months of age.

### Household WASH risk factors.

Each household with a child enrolled in the MAL-ED study was administered a socioeconomic survey adapted from questions used by the Demographic and Health Surveys.^20^ It was administered at 6, 12, 18, and 24 months of age for the children enrolled. The survey included questions related to water (source type, continuity of supply, point-of-use treatment, and collection), sanitation access (type of facility and sharing behavior with other households), hygiene behaviors (hand washing activities and use of toilet paper), and household characteristics (floor type, roofing and wall materials, number of rooms, years of tenancy, electricity, etc.). A household water connection was considered the most hygienic option in this study as these households were less likely to store water, and therefore, there was a lower recontamination risk of the stored water. The pour flush toilet was the most hygienic option according to the Joint Monitoring Program that classifies a flush or pour flush toilet to a septic tank as improved.^21^ A hygiene index variable score was calculated as a cumulative score from the following four questions: 1) Do you wash your hands after helping your child defecate? 2) Do you wash your hands before preparing food? 3) Do you wash your hands after going to the bathroom? and 4) Do you use toilet paper? The hygiene index score had three levels with good indicating the interviewee answered all questions as always practicing the hygienic behaviors; intermediate indicated that for one of the four questions the interviewee only sometimes practiced the hygienic behavior; and poor indicated that for two or more questions the interviewee only sometimes practiced the hygienic behavior. A wealth index was developed based on a sum of different possessions owned in each household giving equal weight to all possessions. Income was not used in the analysis because of the low variability within the community. An overall socioeconomic status index previously developed and validated for the MAL-ED cohort (the WAMI score^22^) was not used because it included water and sanitation variables that were considered key to our hypothesis of interest. Other variables of interest recorded were head-of-household and maternal education, monthly income level (in Soles), and crowding. Breastfeeding status was recorded alongside these variables by a separate questionnaire to characterize exclusive, mixed, and fully weaned breast milk intake.

In addition to the MAL-ED data described earlier, water storage practices were more comprehensively characterized during a community-wide census administered twice, in 2010 and 2012. The questions administered were locally specific and had been previously shown to relate to the risk of diarrheal disease in the study community.^18^ The variables of interest include the total volume of water stored in the household based on the types of containers used for storage and lid type (with or without lids). Questions were recorded by observation from trained field workers who were well acquainted with the local practices of water storage. The census that was administered closest to the child’s birth date was used to represent the water storage variables of the household.

### Stool collection and fecal marker assays.

Stool samples were collected (without fixatives) by field workers on a monthly basis until 12 months of age and then at 15, 18, 21, and 24 months. Children were followed twice weekly for active surveillance for diarrhea and illness. Before stool testing all samples were stored at −70°C. Stool samples were analyzed in parallel for MPO (Alpco, Salem, NH), NEO (GenWay Biotech, San Diego, CA), and AAT (Biovendor, Chandler, NC) as previously described.^8^ All fecal markers are considered stable in stool specimens and resistant to degradation in the intestinal lumen.

### Urine collection and dual sugar test.

The urinary lactulose to mannitol (L:M) dual sugar test has been used most widely to assess intestinal barrier function and identify altered permeability (lactulose) and malabsorption (mannitol).^23^ The dual sugar permeability test was administered under the MAL-ED protocol to each infant at 3, 6, 9, and 15 months of age.^8^ Urine aliquots were stored at −70°C before measurement of lactulose and mannitol concentrations by liquid chromatography-tandem mass spectrometry (LC-MS/MS).^24^ The disaccharide solution was administered after stool sampling was complete to avoid inaccurate protein measurements in stool because of dilution from the watery stool caused by the L:M test.

### Serum collection and plasma markers.

Alpha-1-acid glycoprotein (AGP),^25^ citrulline (CIT),^3^ kynurenine (KYN), tryptophan (TRY) and the kynurenine to tryptophan (KT) ratio^26,27^ were measured in plasma. AGP is a marker of systemic inflammation that was expected to be elevated by EE, and CIT, KYN, and TRY have been proposed as potential EE biomarkers.^11^ Serum CIT is associated with overall mucosal function^28^ and it is reduced in villus atrophy syndrome which has decreased epithelial cell surface area.^10^ KYN, TRY, and KT ratio are markers for T-cell regulated immune responses where the KT ratio represents the amount of KYN synthesized from TRY and TRY depletion is essential for immunosuppressive activity.^26,27^ The KYN and TRY concentrations were analyzed using LC-MS/MS as per Kosek et al.^11^

### Data analysis.

Longitudinal analyses were conducted on the entire sample enrolled up to 24 months of age. The primary outcomes of fecal, urine, and plasma markers were each log-transformed for normality. Only non-diarrheal stool samples were included in the analysis, and fecal markers were mean averaged over 3 months following the age of interest (e.g., months 6, 7, and 8 were mean averaged for the 6-month time point) to reduce the variability within individual. These biomarker averages were then age-matched to the socioeconomic survey data with WASH and household independent variables at the 6-, 12-, 18-, and 24-month time points. The urine and serum markers were similarly age-matched to the independent variables. The relationships between WASH variables and the EE markers were explored using mixed-effects linear regression, with random slopes for age and age-squared and random intercepts specified at the child level to account for within-child clustering. Different mixed models were explored for each biomarker but results remained consistent, and the same analysis is presented here for clarity. The final multivariate mixed-effects models with autoregressive covariance and a random intercept for each child were selected by minimizing the Akaike Information Criterion^29^ based on restricted maximum likelihood estimation. The models were adjusted for age, season, breastfeeding, maternal education, and wealth index. An available-data analysis was used and data were missing at random, therefore, the likelihood-based modeling approach of mixed models was deemed appropriate. To assess model fit, the intraclass correlation (ICC) was used to determine if there was greater variability within than between individuals. In the case of a low ICC (less than 0.10), a multivariate regression model was run to determine the R-squared and adjusted R-squared values of each model to assess the model fit. Data analyses were performed in Stata version 12.1 (College Station, TX).

### Ethics statement.

All data presented in this analysis was collected as part of the Peru site MAL-ED cohort and was approved by institutional review boards from Johns Hopkins Bloomberg School of Public Health (Baltimore, MD) and Asociación Benéfica Proyectos de Informática, Salud, Medicina, y Agricultura (A.B. PRISMA), Lima, Peru.

## RESULTS

A total of 303 children were enrolled from the catchment area and 270 children remained in the study for the 6-month baseline survey with WASH household characteristics. Between each 6-month sampling period, until the children were 24 months of age, the lost to follow up ranged between 7.0% and 11.6% (Table 1). After merging the water storage variables, a total of 258 children in the cohort were included for the analysis. Table 1 describes the WASH variables from each 6-month survey. The WASH variables that were the most time-varying in the population with at least one change reported by a household over the 24-month study included the following: 1) type of sanitation facility used by the household (63.9% of population); 2) household hygiene score (60.5% of the population); and 3) drinking water source option used by the household (55.5% of the population).

**Table 1:** Water, sanitation, hygiene (WASH) and household socioeconomic characteristics of children enrolled in MAL-ED Peru site at 6, 12, 18, and 24 months of age

WASH household characteristic	6 months	12 months	18 months	24 months
	*N* = 270 (*n*)	*N* = 241	*N* = 213	*N* = 198
Type of toilet facility that households usually use (%)				
Flush to septic tank	14.8 (40)	12.0 (29)	12.7 (27)	22.7 (45)
No facility/bush/field or bucket toilet	15.2 (41)	16.6 (40)	18.3 (39)	12.6 (25)
Pit latrine without flush	58.2 (157)	58.9 (142)	56.8 (121)	51.5 (102)
Flush to piped sewer system	1.8 (5)	3.7 (9)	5.2 (11)	8.1 (16)
Flush to pit latrine	1.1 (3)	2.1 (5)	1.4 (3)	2.5 (5)
Flush to somewhere else	6.3 (17)	4.2 (10)	3.3 (7)	1.5 (3)
Type of flooring material (%)				
Cement	21.5 (58)	22.8 (55)	23.6 (50)	27.3 (54)
Dirt	73.0 (197)	69.7 (168)	68.9 (146)	67.7 (134)
Wood	5.6 (15)	7.1 (17)	7.6 (16)	5.1 (10)
Tile	–	0.4 (1)	–	–
Drinking water source (%)				
Piped into dwelling	25.2 (68)	21.6 (52)	22.1 (47)	23.2 (46)
Piped into yard/plot	19.3 (52)	17.8 (43)	17.8 (38)	16.7 (33)
Public tap/stand pipe	4.8 (13)	8.3 (20)	3.3 (7)	1.5 (3)
Tube well or borehole	41.5 (112)	41.1 (99)	42.7 (91)	43.4 (86)
Protected well	1.1 (3)	0.8 (2)	1.9 (4)	3.5 (7)
Unprotected well	3.0 (8)	4.6 (11)	4.7 (10)	6.6 (13)
Surface water	1.1 (3)	2.1 (5)	0.5 (1)	0.5 (1)
Total volume of stored water per capita in liters (reported) (mean)	16.9 (237)	17.3 (215)	17.4 (188)	17.2 (175)
HH uses chlorine to treat their water (%)				
No	85.9 (232)	89.2 (215)	88.3 (188)	82.3 (163)
Yes	14.1 (38)	10.8 (26)	11.7 (25)	17.7 (35)
Continuity of piped water supply (%)				
Continuous	13.0 (35)	17.4 (42)	12.2 (26)	12.6 (25)
Sometimes interrupted	87.0 (235)	82.6 (199)	87.8 (187)	87.4 (173)
Toilet facility is shared:				
No	73.7 (199)	74.7 (180)	79.8 (170)	76.3 (151)
Yes	26.3 (71)	25.3 (61)	20.2 (43)	23.7 (47)
Number of members per household (mean)	6.3 (270)	5.9 (236)	5.8 (213)	5.7 (198)
Household location of cooking activities:				
Inside the house	72.5 (195)	73.0 (176)	80.6 (170)	73.7 (146)
Outside the house	25.3 (68)	25.3 (61)	18.5 (39)	20.7 (41)
Both inside and outside the house	2.2 (6)	1.7 (4)	1.0 (2)	5.6 (11)
Hygiene score				
4	66.7 (180)	68.9 (166)	62.3 (132)	63.6 (126)
3	14.8 (40)	14.1 (34)	14.2 (30)	17.7 (35)
0–2	18.5 (50)	17.0 (41)	23.6 (50)	18.7 (37)
Wealth index (3–18) (mean, 95% CI)	9.5 (9.2, 9.8)	9.4 (9.1, 9.8)	9.3 (9.0, 9.7)	9.9 (9.5, 10.3)
Duration of time family has lived in home (%)				
Less than 1 year	28.5 (77)	20.8 (50)	20.7 (44)	20.3 (40)
Between 1 and 5 years	33.0 (89)	41.9 (101)	36.2 (77)	42.1 (83)
Between 5 and 10 years	16.7 (45)	19.5 (47)	24.9 (53)	18.8 (37)
Between 10 and 20 years	12.2 (33)	8.3 (20)	8.9 (19)	13.7 (27)
More than 20 years	9.6 (26)	9.5 (23)	9.4 (20)	5.1 (10)
Maternal education in years (mean)	7.8 (268)	7.8 (237)	7.9 (211)	7.6 (197)
Breastfeeding				
Mixed	98.6 (216)	96.8 (209)	58.7 (122)	19.0 (37)
Weaned	0.9 (2)	3.2 (7)	41.4 (86)	81.0 (158)

MAL-ED = Etiology, Risk Factors, and Interactions of Enteric Infections and Malnutrition and the Consequences for Child Health and Development Project; WASH = water, sanitation and hygiene.

**Table 2:** Water storage variables from community census with children enrolled in MAL-ED Peru site

Water storage variable	*N*	Mean	SD
Total volume of stored water in the HH (L)	258	99.7	92.9
Option 1 (reported)			
Total volume of stored water in the HH (L)	257	94.6	90.7
Option 2 (summation by lid type)			
No lid: Total volume of stored water in the HH (L)	257	50.3	72.3
Provisional lid: Total volume of stored water in the HH (L)	257	7.6	47.1
Secured lid: Total volume of stored water in the HH (L)	257	36.7	29.5
Total volume of stored water in the HH per capita	258	16.5	15.1
Option 1 (reported)			
Total volume of stored water in the HH per capita	257	15.7	14.6
Option 2 (summation by lid type)			
No lid: Total volume of stored water in the HH per capita	257	8.3	12.1
Provisional lid: Total volume of stored water in the HH per capita	257	1.1	6.7
Secured lid: Total volume of stored water in the HH per capita	257	6.1	4.8
Percent of water stored with no lid	256	37.2	35.2
Percent of water stored with provisional lid	256	5.6	25.2
Percent of water stored with secured lid	256	54.9	36.4
Minimum volume of container used for water storage (L)	204	12.2	8.0
Option 1 (reported directly)			
Minimum volume of container used for water storage (L)	257	12.4	7.4
Option 2 (extracted from data)			
Minimum volume of container used for water storage per capita (L)	204	2.3	1.9
Option 2 (extracted from data)			
Minimum volume of container used for water storage per capita (L)	257	2.2	1.8
Option 2 (reported directly)			

MAL-ED = Etiology, Risk Factors, and Interactions of Enteric Infections and Malnutrition and the Consequences for Child Health and Development Project; SD = standard deviation.

The fecal marker analyte results from asymptomatic stool samples collected at the 6-, 12-, 18-, and 24-month time points and averaged with the subsequent 2 months resulted in 889 observations for MPO, 892 observations for NEO, and 877 observations for AAT. The median concentration for MPO, NEO, and AAT all decreased across the 6-, 12-, 18-, and 24-month time points as shown in Supplemental Table 1.

### Associations between sanitation variables and EE markers.

The community had three main categories for the sanitation facility used by a household: a pour flush toilet in or near the house that flushes to a septic tank onsite (14.8%, *N* = 40), no access to a sanitation facility and instead openly defecated or used a bucket toilet (15.2%, *N* = 41) and pit latrines located outside the home (58.2%, *N* = 157) (Table 1). Shared toilet facilities where a household reported two or more families using the same toilet or latrine was reported in 26.3% of the population (*N* = 71) at the 6-month baseline survey. If families shared their sanitation facilities, there was an average of 2.1 families using the same toilet or latrine. In unadjusted analyses, the households that had either unimproved option of no facility or a pit latrine when compared with the flush toilet both had lower MPO concentrations (−0.34 log [95% CI: −0.61, −0.08] and −0.21 log [95% CI: −0.42, 0.00]) (Table 3). Meanwhile, AGP concentration was higher for households that had no toilet facility versus those with a flush toilet to a septic tank (0.26 log, 95% CI: 0.09, 0.43) (Supplemental Table 2). For households that shared sanitation facilities compared with those that did not share, both the MPO concentration (+0.16 log MPO [95% CI: 0.00, 0.33]) (Table 3) and the KT ratio (+0.18 log KT ratio [95% CI: 0.02, 0.33]) (Supplemental Table 2) were higher. In the fully adjusted models, significant relationships were found for MPO where households with no sanitation facility compared with those with a pour flush toilet had −0.43 log MPO (95% CI: −0.74, −0.13) (Table 4) and the KT ratio where households with a pit latrine without a flush compared with those with a pour flush toilet had +0.10 log KT ratio (95% CI: 0.05, 0.38) (Supplemental Table 3). When the significance level was adjusted for multiple comparisons using the Bonferroni method, the sanitation variable that remained significant was the type of toilet facility that households use in the multivariate MPO model (Table 4).

**Table 3:** Unadjusted mixed models for WASH household characteristics with EE biomarkers in stool and urine (for EE biomarkers in plasma see Supplemental Table 2)

WASH household characteristic	MPO (log [ng/mL])	NEO (log [nmol/L])	AAT (log [mg/g])	L:M
	β (95% CI)	β (95% CI)	β (95% CI)	β (95% CI)
	*N* = 269, *n* = 884	*N* = 269, *n* = 887	*N* = 268, *n* = 871	*N* = 270, *n* = 732
Sanitation				
Type of toilet facility that households usually use				
Flush toilet to septic tank	Ref	Ref	Ref	Ref
No facility/bush/field or bucket toilet	−0.34 (−0.61, −0.08)†	−0.07 (−0.28, 0.14)	−0.07 (−0.32, 0.19)	−0.17 (−0.48, 0.15)
Pit latrine without flush	−0.21 (−0.42, 0.00)*	−0.06 (−0.23, 0.11)	0.09 (−0.12, 0.29)	0.01 (−0.24, 0.26)
Flush toilet to piped sewer system	−0.33 (−0.72, 0.06)	0.05 (−0.26, 0.36)	−0.17 (−0.56, 0.21)	−0.08 (−0.64, 0.48)
Flush toilet to pit latrine	−0.09 (−0.65, 0.47)	0.05 (−0.39, 0.50)	0.19 (−0.39, 0.76)	−0.62 (−1.36, 0.11)
Flush toilet to somewhere else	−0.29 (−0.68, 0.11)	−0.03 (−0.35, 0.28)	0.28 (−0.11, 0.68)	−0.24 (−0.66, 0.18)
Toilet facility is shared				
No	Ref	Ref	Ref	Ref
Yes	0.16 (0.00, 0.33)*	0.05 (−0.09, 0.18)	0.13 (−0.03, 0.30)	0.02 (−0.18, 0.21)
Water				
Drinking water source				
Piped into dwelling	Ref	Ref	Ref	Ref
Piped into yard/plot	0.29 (0.07, 0.52)*	0.28 (0.09, 0.46)†	0.01 (−0.22, 0.24)	−0.07 (−0.33, 0.19)
Public tap/stand pipe	0.16 (−0.19, 0.51)	0.01 (−0.27, 0.29)	0.07 (−0.28, 0.42)	0.38 (−0.002, 0.77)*
Tube well or borehole	0.21 (0.01, 0.40)*	0.16 (0.005, 0.31)*	0.06 (−0.13, 0.25)	0.03 (−0.19, 0.25)
Protected well	0.04 (−0.54, 0.62)	−0.18 (−0.65, 0.28)	−0.26 (−0.83, 0.31)	−0.25 (−1.18, 0.68)
Unprotected well	0.64 (0.27, 1.02)‡	0.32 (0.03, 0.62)*	0.05 (−0.31, 0.41)	0.26 (−0.25, 0.76)
Surface water	−0.05 (−0.78, 0.67)	−0.08 (−0.65, 0.50)	−0.20 (−0.89, 0.49)	−0.31 (−1.05, 0.43)
Total volume of stored water in the HH per capita (reported) in quartiles				
Q1	Ref	Ref	Ref	Ref
Q2	−0.006 (−0.23, 0.22)	−0.08 (−0.26, 0.10)	−0.04 (−0.26, 0.19)	−0.03 (−0.30, 0.24)
Q3	−0.21 (−0.44, 0.02)	−0.14 (−0.33, 0.04)	−0.02 (−0.24, 0.21)	−0.15 (−0.41, 0.11)
Q4	−0.08 (−0.30, 0.15)	0.05 (−0.13, 0.23)	0.11 (−0.11, 0.34)	−0.09 (−0.35, 0.17)
HH uses chlorine to treat their water				
No	Ref	Ref	Ref	Ref
Yes	−0.10 (−0.31, 0.11)	0.02 (−0.15, 0.19)	−0.04 (−0.25, 0.17)	−0.14 (−0.40, 0.11)
Continuity of piped water supply				
Continuous	Ref	Ref	Ref	Ref
Sometimes interrupted	0.37 (0.16, 0.57)‡	0.12 (−0.04, 0.28)	0.23 (0.03, 0.43)*	−0.16 (−0.40, 0.08)
Hygiene				
Hygiene score				
Always	Ref	Ref	Ref	Ref
Most of the time	−0.04 (−0.24, 0.17)	−0.10 (−0.26, 0.06)	0.08 (−0.12, 0.28)	−0.01 (−0.25, 0.23)
Sometimes	0.08 (−0.11, 0.27)	−0.06 (−0.21, 0.09)	−0.04 (−0.23, 0.14)	0.21 (−0.02, 0.43)
Household				
Type of flooring material:				
Cement	Ref	Ref	Ref	Ref
Dirt	−0.07 (−0.25, 0.11)	−0.09 (−0.24, 0.05)	−0.15 (−0.32, 0.03)	−0.04 (−0.25, 0.17)
Wood	−0.07 (−0.41, 0.27)	−0.28 (−0.55, −0.01)*	−0.38 (−0.71, −0.06)*	−0.04 (−0.45, 0.37)
Number of household members in quartiles	−0.02 (−0.06, 0.008)	−0.01 (−0.04, 0.01)	−0.02 (−0.05, 0.01)	0.02 (−0.01, 0.06)
Household location of cooking activities:				
Inside the house	Ref	Ref	Ref	Ref
Outside the house	−0.20 (−0.37, −0.02)*	−0.09 (−0.23, 0.05)	−0.30 (−0.46, −0.13)‡	0.04 (−0.16, 0.23)
Both inside and outside the house	−0.25 (−0.73, 0.23)	0.25 (−0.13, 0.63)	0.44 (−0.001, 0.88)*	0.60 (0.01, 1.19)*
Wealth index in quartiles				
Q1	Ref	Ref	Ref	Ref
Q2	−0.10 (−0.32, 0.13)	0.01 (−0.16, 0.19)	−0.12 (−0.35, 0.10)	−0.12 (−0.35, 0.11)
Q3	−0.12 (−0.31, 0.06)	0.02 (−0.12, 0.17)	0.07 (−0.12, 0.25)	−0.20 (−0.44, 0.04)
Q4	−0.06 (−0.25, 0.13)	–	−0.01 (−0.20, 0.18)	−0.37 (−0.61, −0.12)†
Duration of time family has lived in home				
Less than 1 year	Ref	Ref	Ref	Ref
Between 1 and 5 years	0.07 (−0.11, 0.26)	−0.005 (−0.16, 0.15)	0.04 (−0.14, 0.23)	−0.004 (−0.22, 0.21)
Between 5 and 10 years	0.02 (−0.20, 0.25)	0.03 (−0.15, 0.20)	0.08 (−0.14, 0.30)	−0.05 (−0.31, 0.22)
Between 10 and 20 years	0.21 (−0.06, 0.48)	0.02 (−0.19, 0.24)	0.15 (−0.11, 0.41)	−0.16 (−0.47, 0.14)
More than 20 years	−0.20 (−0.49, 0.09)	−0.17 (−0.40, 0.06)	−0.11 (−0.39, 0.17)	−0.05 (−0.37, 0.27)
Maternal education (years)				
Low	Ref	Ref	Ref	Ref
High	0.15 (0.00, 0.31)*	0.15 (0.02, 0.27)*	0.13 (−0.02, 0.28)	0.02 (−0.16, 0.19)
Child				
Child age (months)	−0.06 (−0.07, −0.05)‡	−0.07 (−0.08, −0.07)‡	−0.07 (−0.08, −0.06)‡	0.04 (0.02, 0.06)‡
Breastfeeding				
Mixed	Ref	Ref	Ref	Ref
Weaned	−0.27 (−0.49, −0.04)*	−0.43 (−0.61, −0.26)‡	−0.55 (−0.77, −0.33)‡	0.36 (−0.39, 1.12)

AAT = alpha-1-antitrypsin; CI = confidence interval; MPO = myeloperoxidase; NEO = neopterin; WASH = water, sanitation, and hygiene.

* Significance at the *P* < 0.05 level.

† Significance at the *P* < 0.01 level.

‡ Significant difference at the *P* < 0.001 level.

**Table 4:** Multivariate mixed-effects models for WASH household characteristics and EE biomarkers in stool and urine. All models adjusted for age, season, breastfeeding, maternal education, and wealth index (for EE biomarkers in plasma see Supplemental Table 3)

	MPO (log [ng/mL])		NEO (log [nmol/L])		AAT (log [mg/g])		LM	
*N*	703		705		691		565	
*n*	194		194		194		194	
	β (95% CI)	*n*	β (95% CI)	*n*	β (95% CI)	*n*	β (95% CI)	*n*
Type of toilet facility households use								
Flush toilet to septic tank	Ref	109	Ref	109	Ref	113	Ref	82
No facility/bush/field or bucket toilet	**−0.43 (−0.74, −0.13)**†	106	−0.06 (**−**0.30, 0.19)	106	−0.07 (**−**0.37, 0.23)	105	−0.28 (**−**0.65, 0.09)	85
Pit latrine without flush	−0.18 (**−**0.42, 0.06)	410	−0.01 (**−**0.21, 0.18)	412	0.07 (**−**0.17, 0.31)	401	−0.03 (**−**0.32, 0.27)	340
Flush toilet to piped sewer system	−0.22 (**−**0.65, 0.22)	29	0.29 (**−**0.06, 0.65)	29	−0.28 (**−**0.74, 0.17)	26	−0.19 (**−**0.85, 0.47)	12
Flush toilet to pit latrine	−0.07 (**−**0.73, 0.59)	11	0.14 (**−**0.39, 0.68)	11	0.03 (**−**0.66, 0.72)	10	−0.39 (**−**1.27, 0.48)	7
Flush toilet to somewhere else	−0.39 (**−**0.83, 0.05)	27	0.04 (**−**0.32, 0.40)	27	0.19 (**−**0.27, 0.64)	26	−0.39 (**−**0.86, 0.07)	29
Drinking water source								
Piped into dwelling	Ref	159	Ref	159	Ref	156	Ref	134
Piped into yard/plot	0.32 (0.06, 0.57)*	144	0.28 (0.07, 0.49)†	144	−0.11 (**−**0.37, 0.15)	140	−0.07 (**−**0.36, 0.22)	128
Public tap/stand pipe	0.40 (**−**0.02, 0.83)	36	−0.03 (**−**0.37, 0.32)	36	0.12 (**−**0.31, 0.56)	35	0.64 (0.14, 1.14)†	35
Tube well or borehole	0.10 (**−**0.14, 0.33)	296	0.20 (0.006, 0.39)*	298	−0.08 (**−**0.31, 0.16)	292	−0.09 (**−**0.36, 0.18)	227
Protected well	0.19 (**−**0.43, 0.81)	12	−0.18 (**−**0.68, 0.33)	12	−0.31 (**−**0.97, 0.35)	11	−0.75 (**−**1.77, 0.26)	5
Unprotected well	0.47 (0.05, 0.88)*	34	0.36 (0.03, 0.70)*	34	−0.16 (**−**0.58, 0.25)	36	−0.11 (**−**0.65, 0.44)	19
Surface water	0.72 (**−**0.30, 1.74)	4	0.60 (**−**0.24, 1.44)	4	−0.30 (**−**1.35, 0.74)	4	−0.51 (**−**1.43, 0.40)	6
Total volume of stored water in the HH in quartiles§								
Q1	Ref	182	Ref	182	Ref	174	Ref	142
Q2	−0.18 (**−**0.43, 0.08)	168	−0.21 (**−**0.41, −0.01)*	169	−0.14 (**−**0.39, 0.10)	170	−0.12 (**−**0.40, 0.17)	122
Q3	−0.33 (**−**0.58, −0.08)†	169	−0.26 (**−**0.46, −0.07)†	170	−0.13 (**−**0.37, 0.12)	165	−0.26 (**−**0.54, 0.01)	154
Q4	−0.26 (**−**0.52, −0.005)*	184	−0.13 (**−**0.33, 0.08)	184	−0.11 (**−**0.36, 0.15)	182	−0.26 (**−**0.55, 0.04)	147
HH uses chlorine to treat their water								
No	Ref	618	Ref	620	Ref	606	Ref	501
Yes	−0.18 (**−**0.43, 0.08)	85	0.06 (**−**0.14, 0.26)	85	0.03 (**−**0.22, 0.28)	85	−0.12 (**−**0.43, 0.19)	64
Continuity of piped water supply								
Continuous	Ref	97	Ref	97	Ref	94	Ref	82
Sometimes interrupted	**0.36 (0.08, 0.63)**†	606	−0.006 (**−**0.23, 0.22)	608	0.20 (**−**0.08, 0.48)	597	0.05 (**−**0.29, 0.39)	483
Practices good hygiene composite score								
Always	Ref	460	Ref	461	Ref	453	Ref	387
Most of the time	−0.04 (**−**0.27, 0.19)	104	−0.08 (**−**0.26, 0.11)	104	0.20 (**−**0.03, 0.44)	100	0.11 (**−**0.17, 0.39)	78
Sometimes	0.11 (**−**0.11, 0.33)	139	0.01 (**−**0.17, 0.20)	140	−0.05 (**−**0.28, 0.18)	138	0.19 (**−**0.08, 0.46)	100
Type of flooring material								
Cement	Ref	166	Ref	167	Ref	166	Ref	125
Dirt	−0.10 (**−**0.31, 0.12)	510	−0.08 (**−**0.26, 0.09)	511	−0.20 (**−**0.42, 0.02)	495	−0.35 (**−**0.61, −0.09)†	419
Wood	−0.03 (**−**0.47, 0.42)	27	−0.23 (**−**0.59, 0.13)	27	−0.30 (**−**0.73,0.13)	30	−0.06 (**−**0.60, 0.48)	21
Household location of cooking activities								
Inside the house	Ref	529	Ref	531	Ref	518	Ref	417
Outside the house	−0.16 (**−**0.35, 0.03)	159	−0.09 (**−**0.24, 0.07)	159	**−0.40 (−0.60, −0.21)**‡	156	0.08 (**−**0.14, 0.31)	134
Both inside and outside the house	−0.42 (**−**0.96, 0.12)	15	−0.09 (**−**0.53, 0.35)	15	0.30 (**−**0.21, 0.82)	17	0.72 (0.13, 1.31)*	14
Wealth Index in quartiles								
Q1	Ref	274	Ref	275	Ref	265	Ref	137
Q2	−0.24 (**−**0.49, 0.01)	88	0.02 (**−**0.19, 0.22)	88	−0.17 (**−**0.43, 0.09)	85	−0.09 (**−**0.35, 0.18)	151
Q3	−0.26 (**−**0.47, −0.04)*	174	−0.04 (**−**0.22, 0.13)	174	−0.07 (**−**0.29, 0.15)	172	−0.28 (**−**0.56, 0.00)*	150
Q4	−0.13 (**−**0.37, 0.12)	167	0.001 (**−**0.19, 0.20)	168	−0.17 (**−**0.41, 0.08)	169	−0.47 (**−**0.78, −0.16)†	127
Maternal education (y)								
Low	Ref	395	Ref	396	Ref	386	Ref	315
High	0.20 (0.02, 0.39)*	308	0.13 (**−**0.02, 0.28)	309	0.11 (**−**0.07, 0.29)	305	0.20 (**−**0.009, 0.40)	250
Child age (months)	−0.06 (**−**0.08, −0.05)‡	703	−0.06 (**−**0.07, −0.05)‡	705	−0.04 (**−**0.06, −0.03)‡	691	0.04 (0.01, 0.06)‡	565
Breastfeeding								
Mixed	Ref	501	Ref	502	Ref	486	Ref	558
Weaned	−0.09 (−0.33, 0.14)	201	−0.42 (**−**0.61, −0.23)‡	202	−0.50 (**−**0.74, −0.26)‡	204	−0.14 (**−**1.11, 0.82)	5
Seasonal effect								
Sine	−0.24 (**−**0.35, −0.14)‡	703	0.06 (**−**0.03, 0.14)	705	0.02 (**−**0.08, 0.13)	691	−0.03 (**−**0.15, 0.09)	565
Cosine	0.11 (0.004, 0.21)*	703	0.02 (**−**0.07, 0.10)	705	−0.01 (**−**0.12, 0.09)	691	−0.09 (**−**0.21, 0.04)	565

AAT = alpha-1-antitrypsin; CI = confidence interval; EE = environmental enteropathy; MPO = myeloperoxidase; NEO = neopterin; WASH = water, sanitation, and hygiene.

* Significance at the *P* < 0.05 level.

† Significance at the *P* < 0.01 level.

‡ Significant difference at the *P* < 0.001 level.

§ Liters of water stored per capita reported directly by the interviewee.

Bold-faced coefficients and confidence intervals signify variables that remained significant after correction for multiple comparisons with the Bonferroni method.

### Associations between water variables and EE markers.

The main drinking water source for households in the study community was a tube well or borehole and in the 6-month survey, this represented 41.5% (*N* = 112) of the population (Table 1). The second most prominent type of drinking water source was a piped water connection to the household for 25.2% (*N* = 68) (Table 1). An intermittent water connection was common with 87.0% of the population reporting interruptions at the 6-month survey (Table 1). The mean total volume of water stored per capita by household was reported to be 16.5 L (standard deviation = 15.1 L) (Table 2). In fully adjusted analyses, households with a piped connection into their yard or plot, there was 0.32 log (95% CI: 0.06, 0.57) and 0.28 log (95% CI: 0.07, 0.49) higher MPO and NEO concentrations, respectively, compared with homes with household piped connections (Table 4). Similarly, households with tube wells or boreholes as their drinking water source had 0.20 log higher NEO concentrations (95% CI: 0.006, 0.39), relative to homes with household piped connections (Table 4). And in fully adjusted analyses for the L:M test, households that used a public tap or stand pipe had higher L:M ratios when compared with homes with household connections (0.64 log, 95% CI: 0.14, 1.14) (Table 4). Those that had intermittent connections had higher MPO concentrations (0.36 log, 95% CI: 0.08, 0.63) than those that had a continuous supply in the fully adjusted multivariate regression models (Table 4). There was also statistical significance for MPO and NEO where homes that stored greater volumes of water had lower MPO concentrations (−0.33 log [95% CI: −0.58, −0.08] for the third quartile and −0.26 log [95% CI: −0.52, −0.005] for the fourth quartile) and lower NEO concentrations (−0.21 log [95% CI: −0.41, −0.01] for the second quartile and −0.26 [95% CI: −0.46, −0.07] for the third quartile) when compared with the quartile with the lowest amount of water stored (Table 4). When the significance level was adjusted for multiple comparisons using the Bonferroni method, the water variable that remained significant was the continuity of piped water supply in the multivariate MPO model (Table 4).

### Associations between hygiene variables and EE markers.

Most of the population (66.7%) reported a hygiene score that indicated they always practiced all hygienic behaviors at the 6-month baseline survey (Table 1). In adjusted analyses, higher TRY and KYN concentrations were reported for those that sometimes practiced hygienic behaviors (0.11 log TRY [95% CI: 0.006, 0.22] and 0.12 log KYN [95% CI: 0.003, 0.23]) when compared with households that always practiced hygienic behaviors (Supplemental Table 3). After adjustment of the significance level with the Bonferroni method, none of the hygiene variables remained significant among the EE biomarkers.

### Associations between household variables and EE markers.

Households in the study community had two main floor types with dirt (73.0%) or cement (21.5%) (Table 1). In fully adjusted analyses, those with dirt floors had a lower L:M ratio (−0.35 log [95% CI: −0.61, −0.09]) when compared with cement floors (Table 4). The location of cooking activities in the households varied by 72.5% inside the house and 25.3% outside the house at the 6-month baseline survey (Table 1). For households with cooking activities performed outside the house, there was lower fecal EE markers for AAT in the fully adjusted model (−0.40 log, 95% CI: −0.60, −0.21) (Table 4). When the significance level was adjusted for multiple comparisons using the Bonferroni method, the only household variable that remained significant was the household location for cooking activities in the multivariate AAT model (Table 4).

## DISCUSSION

We present an in-depth analysis of WASH conditions in a prospective longitudinal study to show associations between water reliability and toilet type with EE biomarkers in a birth cohort for the first 24 months of life. After adjusting for potentially confounding covariates, the hypothesized water pathway showed higher EE for less protected drinking water sources (+0.32 log MPO and +0.28 log NEO for water piped to a yard or plot, and +0.20 log NEO for water from a tube well compared with a household piped water connection), lower EE as the water quantity stored per capita increased (−0.33 log MPO for third quartile, −0.26 log MPO for fourth quartile, −0.21 log NEO for second quartile, and −0.26 log NEO compared with the first quartile of amount of water stored), and higher EE for households that had a water supply that experienced interruptions (+0.36 log MPO). The hypothesized sanitation pathway also showed lower EE for households that did not have access to a toilet facility, and therefore, defecated in places thought to be a greater distance from their household living environments (−0.43 log MPO). Higher KT ratios were also found for households with unimproved pit latrines when compared with homes with improved flush toilets.

Even among a relatively contained community with many shared infrastructure characteristics, we found significant associations in the gut health of children from homes that used different types of toilet facilities and drinking water that was continuous versus intermittent. The longitudinal study design captured the changes in the toilet facilities used by households and accounted for these changes over time in the longitudinal study in relation to the development of EE. Interestingly, the overall environmental contamination caused by open defecation did not nullify the differences between households even though households in the study community were in relative proximity to one another at the village level.

The finding that the pour flush toilet sanitation option, which meets the definition for improved sanitation by the JMP was associated with higher fecal markers for EE compared with the unimproved option of no facility, is important for this study setting. We hypothesize this finding is attributable to the common occurrence of fecal matter overflowing from the on-site sanitation storage pits and contaminating the surrounding household environment.^30^ The pour flush toilets were typically located in closer proximity to the households than the unimproved sanitation options and may have exposed children in these households to more pathogenic material.^31^

This study found evidence for increased EE fecal markers (MPO) in children from households that had interruptions in their water supply. Interruptions in water supply may force a household to use a less protected water sources or may contaminate the piped water supply from a loss of pressure and allow environmental waters to enter the pipes, which are often contaminated where sanitary improvements are lacking.^32^ This finding supports the assertion that improved drinking water sources will not make meaningful contributions to public health if these systems are subject to poor reliability.^33^ A low availability of water stored in liters per capita was recorded in the households of the study community with an average of 16.5 L per capita. This is far below the recommended quantity of 50 L per person per day to meet basic health needs for drinking, cooking, and hygiene.^34^ This study found an inverse relationship with the amount of water stored and EE fecal markers (MPO and NEO), and although not significant after Bonferroni correction, this suggests that a greater quantity of water available in the home improves the gut health.

Households that performed their cooking activities outside had lower EE as measured by AAT concentrations, a marker for nutrient wasting. This highly significant finding showed the potential protective effect that solar inactivation may have for open air kitchens on child gut health. We hypothesize that there is a greater risk of exposure to fecal pathogens in a household when cooking and water activities are performed in an enclosed, dark, and humid environment. By contrast, homes that cook outside may benefit from solar inactivation of fecal pathogens^35^ or wash out of fecal contamination following rain events.

This study was the first to compare associations between the WASH characteristics across eight markers for intestinal inflammation, permeability, and nutrient absorptive capacity as standalone determinants for the progression toward EE. Fecal MPO showed the greatest sensitivity to WASH factors, followed by fecal NEO. The variability explained by the WASH variables alone (i.e., without age and breastfeeding) in the model for MPO amounted to only 2.1% (adjusted r-squared), the most of any biomarker analyzed. Most of the variability in the model was accounted for by the age and breastfeeding variables which have been identified as common factors influencing the concentration of the fecal EE biomarkers.^13^ The low variability accounted for by the WASH variables in the model may also be influenced by how enteropathogen exposures are strongly dependent on age with increased exposure occurring as children become more mobile around 24 months of age.^12^ There is a need to assess the utility of potential biomarkers for EE within communities of varying levels of WASH characteristics because a clear gold standard is not yet defined. This analysis provides support to continue using MPO in exploratory WASH studies.

This study has several important limitations. First, data collection on the water storage variables only occurred at the baseline of the study from the community census. Given the importance of water storage in the community, it would have been preferred to align the community census variables for water storage with the longitudinal data collection of the MAL-ED socioeconomic survey variables and the collection of the EE markers. Second, information on fecal matter storage and treatment was not available. The main sanitation variable used in this analysis was the type of toilet facility and often this does not guarantee safe and sanitary removal of fecal matter from the household environment. Last, the heterogeneity of the floor type variable was not captured by the socioeconomic survey. Often the homes in this community that identified cement floors as their floor type had cement in the entrance area, whereas at the back of the house, where the cooking and washing activities take place, is often dirt.^36^ This may have misclassified homes with mostly dirt floors as having cement and underestimated the risk of exposure to fecal pathogens that elevate the EE markers through the floor pathway. The strengths of the study are the longitudinal design with monthly measurements for the EE fecal markers from birth that were averaged over multiple months to closely track the trajectory of these markers. The concentration of the fecal biomarkers is highly variable between samples from an individual child^13^ and this averaging reduces that variability. The questionnaire that was administered every 6 months also provided a close monitoring of the WASH characteristics of the home. The time-varying sanitation and water variables also demonstrated the importance of a longitudinal study design in low-income communities with high fecal contamination where these variables are not static. And last, the community census provided detailed information on water storage which was widely practiced throughout the community. These strengths combined reduced potential biases to understand the relationships between the WASH characteristics of the household and the development of EE in this cohort.

Water and sanitation conditions were associated with fecal markers for EE in this peri-urban community of Iquitos, Peru. The results provide preliminary evidence for the hypothesis that children less than 24 months of age living in unsanitary conditions will have elevated levels of fecal markers for gut inflammation. Our findings show that WASH factors, in particular the type of toilet and interruptions in water supply, have demonstrable explanatory power for more proximal indicators of EE. Future studies are needed to examine the usefulness of these fecal markers in diverse settings where there is in-depth understanding of the WASH and household characteristics leading to increased contamination and exposure to fecal pathogens.

## Supplementary Material

Supplemental Tables
