# Supplementary material for: A Longitudinal Study of Household Water, Sanitation, and Hygiene Characteristics and Environmental Enteropathy Markers in Children Less than 24 Months in Iquitos, Peru
Source: Am J Trop Med Hyg. 2018 Feb 12;98(4):995–1004. doi: 10.4269/ajtmh.17-0464 (PMC5928816; doi:10.4269/ajtmh.17-0464)
Supplement: Supplementary file 1 [file tpmd170464.SD1.pdf]

Supplemental Table 1

Median concentrations of fecal markers of environmental enteropathy (EE)—myeloperoxidase (MPO), neopterin (NEO), and alpha-1-antitrypsin (AAT)

	MPO (ng/mL)	NEO (nmol/L)	AAT (mg/g)
6 months	14,363	3,782	0.53
12 months	9,047	2,758	0.44
18 months	6,215	1,623	0.35
24 months	4,002	1,037	0.19

Supplemental Table 2

Unadjusted mixed models for WASH household characteristics with plasma markers for EE

WASH household characteristic	AGP (log [mg/dL])	CIT (log [µmol/L])	TRY (log [µmol/L])	KYN (log [µmol/L])	KT ratio
	β (95% CI)	β (95% CI)	β (95% CI)	β (95% CI)	β (95% CI)
	N = 236, N = 439	N = 260, N = 622	N = 260, N = 622	N = 260, N = 622	N = 260 N = 622
Sanitation					
Type of toilet facility that households usually use					
Flush toilet to septic tank	Ref	Ref	Ref	Ref	Ref
No facility/bush/field or bucket toilet	**0.26 (0.09, 0.43)†**	−0.001 (−0.10, 0.10)	−0.04 (−0.16, −0.07)	−0.03 (−0.15, 0.10)	0.01 (−0.09, 0.12)
Pit latrine without flush	0.02 (−0.12, 0.16)	0.04 (−0.04, 0.12)	−0.05 (−0.15, 0.05)	−0.01 (−0.11, 0.09)	0.04 (−0.05, 0.12)
Flush toilet to piped sewer system	–	−0.05 (−0.20, 0.09)	−0.10 (−0.27, 0.06)	−0.08 (−0.25, 0.10)	0.04 (−0.11, 0.19)
Flush toilet to pit latrine	−0.07 (−0.53, 0.39)	**−0.22 (−0.42, −0.008)***	−0.09 (−0.32, 0.15)	−0.22 (−0.47, 0.04)	−0.14 (−0.35, 0.08)
Flush toilet to somewhere else	0.20 (−0.05, 0.45)	0.07 (−0.08, 0.22)	−0.10 (−0.28, 0.07)	0.09 (−0.10, 0.27)	**0.18 (0.02, 0.33)***
Toilet facility is shared					
No	Ref	Ref	Ref	Ref	Ref
Yes	−0.07 (−0.18, 0.05)	−0.01 (−0.08, 0.05)	−0.007 (−0.08, 0.07)	0.06 (−0.02, 0.14)	**0.07 (0.0004, 0.13)***
Water					
Drinking water source					
Piped into dwelling	Ref	Ref	Ref	Ref	Ref
Piped into yard/plot	−0.04 (−0.19, 0.11)	0.07 (−0.01, 0.16)	0.07 (−0.03, 0.17)	0.008 (−0.10, 0.11)	−0.06 (−0.14, 0.03)
Public tap/stand pipe	0.03 (−0.20, 0.26)	0.05 (−0.08, 0.19)	0.02 (−0.13, 0.18)	−0.07 (−0.23, 0.09)	−0.10 (−0.24, 0.04)
Tube well or borehole	−0.01 (−0.13, 0.110	**0.09 (0.01, 0.16)***	0.04 (−0.04, 0.12)	0.04 (−0.05, 0.13)	−0.002 (−0.08, 0.07)
Protected well	0.09 (−0.31, 0.48)	−0.002 (−0.21, 0.21)	−0.03 (−0.27, 0.21)	−0.07 (−0.33, 0.19)	−0.05 (−0.27, 0.17)
Unprotected well	0.02 (−0.26, 0.29)	0.07 (−0.07, 0.22)	0.01 (−0.15, 0.18)	−0.04 (−0.22, 0.14)	−0.07 (−0.21, 0.09)
Surface water	0.38 (−0.09, 0.85)	−0.15 (−0.45, 0.14)	−0.14 (−0.47, 0.19)	0.18 (−0.18, 0.53)	**0.33 (0.03, 0.63)***
Total volume of stored water in the HH per capita (reported)					
Q1	Ref	Ref	Ref	Ref	Ref
Q2	−0.04 (−0.19, 0.11)	−0.04 (−0.13, 0.05)	0.002 (−0.10, 0.10)	0.03 (−0.08, 0.14)	0.02 (−0.07, 0.12)
Q3	−0.13 (−0.27, 0.02)	−0.02 (−0.11, 0.07)	0.009 (−0.09, 0.11)	0.10 (−0.01, 0.20)	**0.09 (−0.001, 0.18)***
Q4	−0.12 (−0.26, 0.02)	0.008 (−0.08, 0.09)	0.01 (−0.08, 0.11)	0.03 (−0.07, 0.14)	0.02 (−0.07, 0.11)
HH uses chlorine to treat their water					
No	Ref	Ref	Ref	Ref	Ref
Yes	0.0003 (−0.15, 0.15)	−0.03 (−0.11, 0.05)	0.05 (−0.05, 0.14)	−0.02 (−0.13, 0.08)	−0.07 (−0.15, 0.02)
Continuity of piped water supply					
Continuous	Ref	Ref	Ref	Ref	Ref
Sometimes interrupted	0.03 (−0.10, 0.17)	0.02 (−0.06, 0.10)	0.06 (−0.03, 0.14)	0.08 (−0.02, 0.17)	0.03 (−0.05, 0.11)
Hygiene					
Hygiene Score:					
Always	Ref	Ref	Ref	Ref	Ref
Most of the time	**0.15 (0.01, 0.28)***	0.07 (−0.009, 0.14)	0.05 (−0.04, 0.14)	0.07 (−0.03, 0.16)	0.02 (−0.06, 0.10)
Sometimes	−0.05 (−0.18, 0.09)	**0.08 (0.001, 0.15)***	0.04 (−0.05, 0.12)	0.06 (−0.04, 0.15)	0.01 (−0.07, 0.09)
Household					
Type of flooring material					
Cement	Ref	Ref	Ref	Ref	Ref
Dirt	0.02 (−0.09, 0.14)	−0.005 (−0.07, 0.06)	−0.008 (−0.08, 0.07)	−0.02 (−0.10, 0.06)	−0.15 (−0.32, 0.03)
Wood	0.18 (−0.03, 0.40)	0.05 (−0.08, 0.18)	−0.11 (−0.26, 0.04)	0.11 (−0.05, 0.27)	**0.22 (0.09, 0.36)***

(continued)

Supplemental Table 2

Continued

WASH household characteristic	AGP (log [mg/dL]) β (95% CI) $N = 236, N = 439$	CIT (log [µmol/L]) β (95% CI) $N = 260, N = 622$	TRY (log [µmol/L]) β (95% CI) $N = 260, N = 622$	KYN (log [µmol/L]) β (95% CI) $N = 260, N = 622$	KT ratio β (95% CI) $N = 260$ $N = 622$
Number of household members in quartiles					
Q1	Ref	Ref	Ref	Ref	Ref
Q2	−0.008 (−0.14, 0.12)	0.01 (−0.06, 0.08)	**0.09 (0.01, 0.17)***	0.08 (−0.01, 0.17)	−0.01 (−0.08, 0.07)
Q3	−0.09 (−0.25, 0.06)	−0.007 (−0.10, 0.08)	0.02 (−0.08, 0.12)	−0.02 (−0.13, 0.09)	−0.04 (−0.13, 0.06)
Q4	0.02 (−0.11, 0.16)	−0.01 (−0.09, 0.08)	0.001 (−0.09, 0.09)	0.05 (−0.04, 0.15)	0.06 (−0.02, 0.14)
Household location of cooking activities					
Inside the house	Ref	Ref	Ref	Ref	Ref
Outside the house	0.05 (−0.07, 0.16)	0.04 (−0.02, 0.11)	0.003 (−0.07, 0.08)	0.001 (−0.08, 0.08)	−0.009 (−0.07, 0.07)
Both inside and outside the house	0.05 (−0.23, 0.34)	0.05 (−0.12, 0.21)	0.07 (−0.11, 0.26)	−0.10 (−0.30, 0.10)	**−0.17 (−0.34, 0.001)***
Wealth index:					
Q1	Ref	Ref	Ref	Ref	Ref
Q2	−0.11 (−0.27, 0.05)	0.05 (−0.04, 0.14)	0.04 (−0.06, 0.14)	−0.03 (−0.14, 0.07)	−0.08 (−0.17, 0.01)
Q3	−0.06 (−0.19, 0.06)	−0.02 (−0.09, 0.05)	0.02 (−0.06, 0.11)	−0.03 (−0.12, 0.06)	−0.06 (−0.13, 0.02)
Q4	−0.11 (−0.24, 0.02)	0.06 (−0.02, 0.13)	0.04 (−0.04, 0.12)	0.04 (−0.05, 0.13)	−0.01 (−0.08, 0.07)
Duration of time family has lived in home					
Less than 1 year	Ref	Ref	Ref	Ref	Ref
Between 1 and 5 years	−0.05 (−0.17, 0.08)	−0.03 (−0.10, 0.04)	0.01 (−0.08, 0.09)	−0.03 (−0.12, 0.06)	−0.03 (−0.11, 0.04)
Between 5 and 10 years	−0.07 (−0.22, 0.07)	0.07 (−0.01, 0.16)	0.06 (−0.04, 0.16)	0.003 (−0.10, 0.11)	−0.05 (−0.14, 0.04)
Between 10 and 20 years	−0.04 (−0.21, 0.13)	−0.02 (−0.12, 0.08)	0.03 (−0.08, 0.15)	−0.06 (−0.18, 0.06)	−0.09 (−0.20, 0.02)
More than 20 years	–	−0.03 (−0.14, 0.08)	−0.05 (−0.18, 0.07)	**−0.14 (−0.28, −0.01)***	−0.09 (−0.20, 0.03)
Maternal education (y)					
Low	Ref	Ref	Ref	Ref	Ref
High	−0.09 (−0.19, 0.02)	0.005 (−0.05, 0.06)	0.02 (−0.04, 0.09)	−0.01 (−0.08, 0.06)	−0.03 (−0.09, 0.03)
Child					
Child age (months)	−0.007 (−0.02, 0.001)	**0.03 (0.03, 0.04)†**	**−0.01 (−0.02, −0.01)†**	**−0.03 (−0.03, −0.02)†**	**−0.01 (−0.02, −0.01)†**
Breastfeeding					
Mixed	Ref	Ref	Ref	Ref	Ref
Weaned	−0.02 (−0.21, 0.18)	−0.05 (−0.15, 0.05)	−0.10 (−0.22, 0.02)	−0.08 (−0.21, 0.04)	−0.0002 (−0.11, −0.11)

AAT = alpha-1-antitrypsin; AGP = alpha-1-acid glycoprotein; CI = confidence interval; EE = environmental enteropathy; KT = kynurenine to tryptophan; KYN = kynurenine; TRY = tryptophan; WASH = water, sanitation, and hygiene.

* Significance at the $P < 0.05$ level.

† Significant difference at the $P < 0.001$ level.

Supplemental Table 3

Multivariate mixed-effects models for WASH household characteristics and plasma markers for EE

	AGP (log [mg/dL])		CIT (log [µmol/L])		TRY (log [µmol/L])		KYN (log [µmol/L])		KT ratio	
N	344		494		494		494		494	
n	175		194		194		194		194	
	β (95% CI)	*n*	β (95% CI)	*n*	β (95% CI)	*n*	β (95% CI)	*n*	β (95% CI)	*n*
Type of toilet facility households use										
Flush toilet to septic tank	Ref	57	Ref	79	Ref	79	Ref	79	Ref	79
No facility/bush/field or bucket toilet	0.18 (−0.04, 0.37)	53	−0.006 (−0.12, 0.11)	71	−0.06 (−0.20, 0.08)	71	−0.003 (−0.15, 0.14)	71	0.04 (−0.08, 0.16)	71
Pit latrine without flush	0.03 (−0.13, 0.19)	203	0.03 (−0.07, 0.12)	284	−0.09 (−0.20, 0.02)	284	0.006 (−0.11, 0.12)	284	**0.10 (0.002, 0.19)***	284
Flush toilet to piped sewer system	0.26 (−0.27, 0.78)	4	0.04 (−0.13, 0.20)	21	−0.10 (−0.29, 0.10)	21	−0.02 (−0.23, 0.18)	21	0.08 (−0.09, 0.26)	21
Flush toilet to pit latrine	−0.11 (−0.66, 0.44)	4	**−0.30 (−0.54, −0.05)***	9	−0.17 (−0.46, 0.12)	9	−0.15 (−0.45, 0.15)	9	0.01 (−0.24, 0.26)	9
Flush toilet to somewhere else	**0.30 (0.02, 0.59)***	17	0.07 (−0.09, 0.23)	23	−0.12 (−0.31, 0.07)	23	0.11 (−0.09, 0.31)	23	**0.22 (0.05, 0.38)**†	23
Drinking water source										
Piped into dwelling	Ref	92	Ref	117	Ref	117	Ref	117	Ref	117
Piped into yard/plot	−0.04 (−0.21, 0.13)	76	0.07 (−0.03, 0.17)	105	0.07 (−0.05, 0.18)	105	−0.02 (−0.15, 0.10)	105	−0.09 (−0.19, 0.01)	105
Public tap/stand pipe	0.11 (−0.18, 0.40)	3	−0.03 (−0.20, 0.13)	25	−0.06 (−0.25, 0.14)	25	−0.09 (−0.29, 0.11)	25	−0.05 (−0.22, 0.12)	25
Tube well or borehole	−0.07 (−0.23, 0.09)	133	0.06 (−0.03, 0.15)	204	−0.02 (−0.12, 0.09)	204	−0.04 (−0.15, 0.07)	204	−0.03 (−0.12, 0.07)	204
Protected well	0.06 (−0.42, 0.54)	5	−0.02 (−0.26, 0.21)	9	−0.17 (−0.44, 0.11)	9	−0.13 (−0.42, 0.16)	9	0.03 (−0.21, 0.27)	9
Unprotected well	−0.04 (−0.37, 0.28)	12	0.04 (−0.12, 0.21)	22	−0.04 (0.23, 0.15)	22	−0.11 (−0.31, 0.09)	22	−0.09 (−0.26, 0.08)	22
Surface water	−0.41 (−1.51, 0.68)	1	**−0.58 (−1.06, −0.09)***	2	−0.50 (−1.06, 0.07)	2	−0.09 (−0.69, 0.50)	2	0.43 (−0.07, 0.92)	2
Total volume of stored water in the HH§						132				
Q1	Ref	87	Ref	129	Ref	129	Ref	129	Ref	129
Q2	−0.06 (−0.22, 0.11)	73	−0.06 (−0.16, 0.03)	108	−0.008 (−0.12, 0.10)	108	−0.007 (−0.13, 0.11)	108	−0.004 (−0.10, 0.10)	108
Q3	−0.10 (−0.26, 0.06)	93	−0.02 (−0.12, 0.07)	125	−0.007 (−0.11, 0.10)		0.05 (−0.06, 0.17)	125	0.05 (−0.04, 0.15)	125
Q4	−0.15 (−0.32, 0.02)	91	0.007 (−0.09, 0.10)	132	0.01 (−0.10, 0.12)		0.01 (−0.10, 0.13)	132	0.002 (−0.10, 0.10)	132
HH uses chlorine to treat their water										
No	Ref	311	Ref	438	Ref	438	Ref	438	Ref	438
Yes	0.05 (−0.15, 0.25)	33	0.009 (−0.09, 0.11)	56	0.10 (−0.02, 0.22)	56	0.06 (−0.07, 0.18)	56	−0.04 (−0.14, 0.07)	56
Continuity of piped water supply										
Continuous	Ref	52	Ref	71	Ref	71	Ref	71	Ref	71
Sometimes interrupted	0.09 (−0.11, 0.28)	292	−0.02 (−0.12, 0.08)	423	0.02 (−0.10, 0.14)	423	0.10 (−0.03, 0.22)	423	0.07 (−0.03, 0.18)	423
Practices good hygiene composite score										
Always	Ref	232	Ref	326	Ref	326	Ref	326	Ref	326
Most of the time	0.14 (−0.02, 0.30)	54	0.08 (−0.005, 0.17)	88	0.08 (−0.02, 0.18)	80	0.06 (−0.05, 0.17)	80	−0.02 (−0.11, 0.07)	80
Sometimes	−0.05 (−0.22, 0.11)	58	0.09 (−0.004, 0.18)	80	**0.11 (0.006, 0.22)***	88	**0.12 (0.003, 0.23)***	88	0.001 (−0.09, 0.10)	88
Type of flooring material										
Cement	Ref	83	Ref	118	Ref	118	Ref	118	Ref	118
Dirt	−0.001 (−0.17, 0.13)	243	−0.008 (−0.09, 0.08)	357	−0.06 (−0.16, 0.04)	357	−0.08 (−0.19, 0.02)	357	−0.02 (−0.11, 0.06)	357
Wood	0.06 (−0.21, 0.33)	18	0.11 (−0.07, 0.28)	19	−0.14 (−0.35, 0.06)	19	0.05 (−0.16, 0.27)	19	**0.21 (0.03, 0.39)***	19
Household location of cooking activities										
Inside the house	Ref	249	Ref	360	Ref	360	Ref	360	Ref	360
Outside the house	0.02 (−0.12, 0.16)	84	0.03 (−0.04, 0.10)	120	0.003 (−0.08, 0.09)	120	0.003 (−0.09, 0.09)	120	0.002 (−0.07, 0.08)	120
Both inside and outside the house	−0.02 (−0.35, 0.31)	11	0.04 (−0.15, 0.22)	14	0.02 (−0.19, 0.24)	14	−0.15 (−0.38, 0.08)	14	−0.17 (−0.36, 0.02)	14
Wealth index										
Q1	Ref	127	Ref	189	Ref	189	Ref	189	Ref	189
Q2	−0.04 (−0.23, 0.15)	40	0.06 (−0.04, 0.16)	60	−0.02 (−0.14, 0.10)	60	−0.04 (−0.16, 0.09)	60	−0.03 (−0.13, 0.07)	60
Q3	−0.03 (−0.18, 0.12)	91	−0.02 (−0.10, 0.07)	118	−0.02 (−0.12, −0.07)	118	−0.07 (−0.17, 0.04)	118	−0.05 (−0.13, 0.04)	118
Q4	−0.02 (−0.19, 0.15)	86	0.06 (−0.03, 0.15)	127	−0.02 (−0.12, 0.09)	127	0.01 (−0.10, 0.12)	127	0.02 (−0.08, 0.11)	127

(continued)

Supplemental Table 3
Continued

	AGP (log [mg/dL])		CIT (log [µmol/L])		TRY (log [µmol/L])		KYN (log [µmol/L])		KT ratio	
N	344		494		494		494		494	
n	175		194		194		194		194	
	β (95% CI)	*n*	β (95% CI)	*n*	β (95% CI)	*n*	β (95% CI)	*n*	β (95% CI)	*n*
Maternal education (y)										
Low	Ref	197	Ref	258	Ref	278	Ref	278	Ref	278
High	−0.04 (−0.16, 0.08)	147	0.01 (−0.06, 0.08)	182	0.03 (−0.05, 0.11)	216	−0.01 (−0.10, 0.07)	216	−0.04 (−0.11, 0.03)	216
Child age (months)	−0.01 (−0.02, 0.003)		**0.04 (0.03, 0.04)**‡				**−0.02 (−0.03, −0.01)**‡		**−0.01 (−0.01, 0.00)***	
		344		494	**−0.01 (−0.02, −0.004)**†	494		494		494
Breastfeeding										
Mixed	Ref	296	Ref	362	Ref	362	Ref	362	Ref	362
Weaned	0.002 (−0.23, 0.23)	47	−0.08 (−0.20, 0.03)	131	−0.09 (−0.22, 0.05)	131	−0.11 (−0.25, 0.03)	131	−0.05 (−0.17, 0.07)	131
Seasonal effect										
Sine	0.002 (−0.08, 0.08)	344	0.01 (−0.03, 0.05)	494	0.02 (−0.03, 0.07)	494	0.02 (−0.03, 0.08)	494	0.003 (−0.04, 0.04)	494
Cosine	−0.02 (−0.10, 0.05)	344	−0.003 (−0.04, 0.04)	494	−0.001 (−0.05, 0.05)	494	−0.01 (−0.06, 0.04)	494	−0.01 (−0.05, 0.03)	494

AGP = alpha-1-acid glycoprotein; CI = confidence interval; EE = environmental enteropathy; KT = kynurenine to tryptophan; KYM = kynurenine; TRY = tryptophan; WASH = water, sanitation, and hygiene.
All models adjusted for age, season, breastfeeding, maternal education, and wealth index.
* Significance at the $P < 0.05$ level.
† Significance at the $P < 0.01$ level.
‡ Significant difference at the $P < 0.001$ level.
§ Liters of water stored per capita reported directly by the interviewee.
